# Occupational health of employees with mental health issues: the role of the psychosocial working conditions and sense of coherence

**DOI:** 10.1007/s00420-025-02154-7

**Published:** 2025-06-19

**Authors:** Anja I. Lehmann, Georg F. Bauer

**Affiliations:** Division Public & Organizational Health, Center of Salutogenesis, Department of Public and Global Health, Epidemiology, Biostatistics and Prevention Institute, University of Zurich, Zurich, Switzerland

**Keywords:** Work ability, Work engagament, Burnout, Mental health, Sense of coherence, Job resources

## Abstract

**Background:**

The high prevalence of mental health issues (MHI) among employees poses significant societal challenges. However, little is known about factors that influence their ability to remain employed, maintain productivity, and thrive at work.

**Objective:**

This study examines differences in occupational health outcomes (burnout, work engagement, and work ability) between employees with and without MHI and the applicability of the Job Demands-Resources model (including job demands, job resources as psychosocial working conditions and sense of coherence (SOC) as a personal resource) among employees with MHI, while particularly controlling for disease-specific factors.

**Methods:**

Pooled data from two measurement waves were analyzed, including employees with current MHI (*N* = 92) and those without MHI (*N* = 877) from German-speaking countries. Mixed-effects models examined relationships between sociodemographic, disease-specific factors, psychosocial working conditions, SOC, and occupational health outcomes.

**Results:**

Employees with MHI showed lower occupational health levels (higher burnout, reduced work ability) than those without. Among employees with MHI, SOC and job resources were significantly associated with all occupational health outcomes, while job demands primarily predicted burnout. Sickness absence correlated negatively with both burnout and work ability. The association between SOC and work ability was stronger for employees with MHI than for those without.

**Conclusions:**

Job resources and SOC play a role for occupational health in employees with MHI. Targeted interventions to strengthen these resources should be prioritized.

## Background

### Introduction

The high prevalence of mental health issues (MHI) among employees has a significant impact on both individuals and organizations. However, research on the workplace experiences of employees with MHI, particularly the factors that enable them to remain, stay productive, and thrive, remains limited. Most MHI studies stem from clinical research, while management and organizational perspectives remain scarce (Follmer and Jones 2018). Earlier occupational health research primarily focused on preventing MHI, but a recent review highlighted the need to examine employees already experiencing MHI in the workplace (van Hees et al. 2022).

Studying MHI in the workplace is crucial for several reasons. First, given their high prevalence, an increasing number of employees must navigate maintaining employability despite MHI (Follmer and Jones 2018). Second, employees with MHI face higher underemployment and lower incomes (Cook 2006). Third, employers are often hesitant to hire individuals with MHI, resulting in lower employment rates (Rüsch et al. 2014). Fourth, MHI contribute to societal costs through reduced workability, reintegration efforts, and financial support needs (Hoffmann and Richter 2020).

Ample evidence shows that employees with MHI both want and are able to work and that employment can enhance their quality of life (Macias et al. 2001; Secker et al. 2001). The lack of work participation is particularly problematic, as work not only provides financial security but also plays a crucial role in social integration, citizenship, and recovery (Dunn et al. 2008). Conversely, unemployment can have detrimental effects on mental health (Dooley et al. 1994).

Recognizing the significance of MHI in the workplace, this study addresses the gap in research on the work experiences of individuals with MHI (Follmer and Jones 2018). Studies on employed individuals with MHI are rare, partly due to the higher unemployment rates among those with MHI (Honkonen et al. 2007). Additionally, most research has focused on return-to-work and absenteeism rather than how employees with MHI can thrive (Andersen et al. 2012; van Hees et al. 2022). There is a lack of focus on mildly ill individuals who remain employed, and few early intervention concepts exist (Baer 2013). Given the societal costs of mental illnesses, which are often mild to moderate (OECD, 2012), this is a critical area of research. Furthermore, previous studies have been descriptive and lacked theoretical grounding and reliable measurement instruments (Follmer and Jones 2018). Building on this gap, the study uses the Job Demands Resources (JD-R) model (Demerouti et al. 2001) to explore:

1. Occupational health outcomes between employees with and without MHI.
2. The association of individual, disease-specific, and psychosocial working conditions with occupational health outcomes in employees with current MHI.

### Job demands and job resources as psychosocial working conditions

In the present study, we used the JD-R model framework (Demerouti et al. 2001) to examine how psychosocial working conditions explain occupational health (burnout, work engagement and work ability) among employees with MHI. Burnout, as the first outcome, is defined as “a state of physical, emotional and mental exhaustion that results from long-term involvement in work situations that are emotionally demanding” (Schaufeli and Greenglass 2001, p. 501). The second outcome, work engagement, is described as a positive, fulfilling, affective-motivational state of work-related well-being that is characterized by vigor, dedication, and absorption (Bakker et al. 2008). Work ability as the third outcome in this study is defined as the capacity to function successfully at work or to achieve expected work goals (Ilmarinen 2006) and its impairment is a predictor for receiving a disability pension (Alavinia et al. 2009).

According to the JD-R model, every workplace includes demands and resources that represent the psychosocial working conditions. One of the main assumptions of the JD-R model is that these psychosocial working conditions have the potential to initiate two distinct psychological processes. Job demands defined as “aspects of the job that require sustained physical or mental effort and are therefore associated with certain physiological and psychological costs” (Demerouti et al. 2001, p. 501) result in a *health-impairing process*, leading to persistent psychological strain and ultimately negative organizational outcomes, such as burnout (Bakker et al. 2005).

Job resources, on the other hand, refer to work characteristics triggering a *motivational process*. Job resources are physical, psychological, social, or organizational factors that aid task completion, reduce job demands, and foster growth and development. They exist at organizational, interpersonal, work organization, and task levels (Demerouti et al. 2001). Resourceful work characteristics can encourage employees to achieve their goals by tapping into their intrinsic and extrinsic motivational potential. Besides the relationship between job resources and motivational outcomes, job resources can also reduce health-impairing processes (Bakker and Demerouti 2017). Therefore, job resources can achieve a dual function: promoting motivational processes (i.e. increasing work engagement and work ability) (Airila et al. 2014) while reducing health impairing ones (i.e. reducing burnout), whereas job demands are primarily associated with health-impairment processes.

### Sense of coherence as a personal resource

The JD-R model has evolved to recognize that work outcomes are influenced not only by environmental factors but also by individual factors. A key extension of the model includes individual factors such as personal resources (Xanthopoulou et al. 2007), with Sense of Coherence (SOC) serving as an example (Vogt et al. 2016). Based on Salutogenic theory SOC is characterized by the degree to which an individual experiences an enduring confidence that (a) stimuli from both external and internal environments are structured, predictable, and explicable, (b) the individual possesses the necessary resources to effectively address the demands imposed by these stimuli, and (c) these demands are perceived as meaningful challenges deserving of investment and effort (Antonovsky 1987). SOC is an important antecedent for work-related outcomes because an individual with a strong SOC might have a greater ability to mobilize and generate resources in his or her workplace than a person with a weak SOC (Jenny et al. 2022). Thus, it can be hypothesized that individuals with a strong SOC are more adept at discerning the nature of the stressors they encounter and effectively choosing suitable resources tailored to the specific situation. It correlates positively with work engagement (Vogt et al. 2016) and negatively with burnout (Johnston et al. 2013), and sickness absence (Kivimäki et al. 1997).

Research on the role of SOC in employees with MHI remains scarce, despite its relevance as a life orientation for coping with stress (Antonovsky 1987). Beyond the work context, SOC has been linked to health, recovery, life satisfaction, and coping in this group (Langeland et al. 2006, 2007).

### Disease-specific factors as control variables

This study examines how JD-R model factors (job demands and resources) and SOC as a personal resource relate to occupational health in employees with MHI. When studying this group, disease-specific factors should be controlled alongside sociodemographics (e.g., age, gender, education, job position, working hours) to account for alternative explanations. They include: (a) Sickness absence: Reflects the acute severity of the disease and predicts long-term negative effects on work ability (Virtanen et al. 2006). (b) Self-stigma: An important predictor for employment among people with MHI (Rüsch et al. 2014). (c) Disclosure: Disclosure of MHI can lead to accommodations but may also result in stigma and discrimination (Peterson et al. 2011). (d) Treatment: Can improve work-related outcomes (Follmer and Jones 2018; Knekt et al. 2011). (e) Duration of disease: Indicates chronicity, which may pose a risk for remaining in the workplace (San et al. 2013).

### Study aim and hypotheses

The aim of this study was to compare occupational health between employees with and without MHI. We hypothesize that employees with current MHI exhibit lower levels of occupational health, highlighting the need for targeted focus for this group. Accordingly, the second aim focused on employees with MHI, examining psychosocial working conditions and SOC in relation to occupational health while controlling for disease-specific and socio-demographic factors.

The hypotheses of the study are as follows:

#### Hypothesis 1:

Employees with current MHI will exhibit lower occupational health ((a) burnout, (b) work engagement, and (c) work ability) compared to those without MHI.

#### Hypothesis 2:

Among employees with current MHI, job resources will be (a) negatively associated with burnout, (b) positively associated with work engagement, and (c) positively associated with work ability.

#### Hypothesis 3:

Among employees with current MHI, job demands will be positively associated with burnout.

#### Hypothesis 4:

Among employees with current MHI, SOC will be (a) negatively associated with burnout, (b) positively associated with work engagement, and (c) positively associated with work ability.

## Methods

### Overall study design and study population

This online self-report survey was part of a larger research project. Employees from various occupational fields in German-speaking countries who worked over 20 h were recruited via a high-quality panel provider (“Bilendi”). Participants received a modest incentive in redeemable points. Data were collected in two waves (April–June 2022 and December 2022–January 2023) and then pooled. The sample aligned with labor force demographics (Vogt et al. 2016), and meta-analyses confirm online panel data reliability (Walter et al. 2019). Participation was voluntary, informed consent was obtained, and data confidentiality was ensured. Ethical approval was not required.

### Measurements

#### Psychosocial working conditions

Job resources were assessed using subscales from the HSE Management Standards Indicator Tool (Cousins et al. 2004), including managerial support (5 items, e.g., “I can rely on my line manager to help me out with a work problem”) and peer support (4 items, e.g., “My colleagues are willing to listen to my work-related problems”). Additionally, 3 items from the SALSA development subscale (Udris and Rimann 1999; e.g., “At work, you can develop your skills”) were used. The overall job resources scale (that covered all 9 items) had a Cronbach’s alpha of α = 0.89.

Job demands were measured with 8 items from the HSE quantitative demands subscale (e.g., “I have to neglect some tasks because I have too much to do.”) and three items from the SALSA qualitative demands subscale (Udris and Rimann 1999; e.g., “It happens that work is too difficult for me.”). All items were rated on a five-point Likert scale (1 = “not true at all” to 5 = “very true”). Cronbach’s alpha was α = 0.87.

#### Sense of coherence

Sense of coherence was assessed using the 13-item short form of the Orientation to Life Scale (SOC-13; Antonovsky 1987). Responses were given on a seven-point Likert scale, with a total score ranging from 13 (weak SOC) to 91 (strong SOC). Cronbach’s alpha was α = 0.88.

#### Work ability

Perceived current work ability was measured with a single item from the Work Ability Index (WAI; Ilmarinen 2006): “How many points would you give your current ability to work?” The scale ranged from 0 (“cannot currently work at all”) to 10 (“work ability at its lifetime best”).

#### Work engagement

Work engagement was assessed using the shortened version of the Utrecht Work Engagement Scale (UWES-6; Schaufeli and Bakker 2003). The scale consists of 6 items rated on a seven-point Likert scale (0 = “never” to 6 = “daily”), e.g., “At my job, I feel strong and vigorous.” Cronbach’s alpha was α = 0.97.

#### Work-related burnout

Work-related burnout was assessed using 7 items from the Copenhagen Burnout Inventory (CBI; Kristensen et al. 2005). Responses were given on a five-point Likert scale (1 = “never” to 5 = “very often”), e.g., “Do you feel burnt out because of your work?”. Cronbach’s alpha was α = 0.91.

#### Mental health status

MHI were measured using a single item in which respondents indicated whether they (0) currently have, (1) have had in the past, or (2) have never had an MHI. The item was dichotomized into 0 = current MHI and 1 = no MHI (combining categories 1 and 2). Self-reported single-items are often used in research (Andersson et al. 2014; Browne et al. 2023; Doherty et al. 2010) as a feasible and sensitive mental health indicator (Reme et al. 2010; Rutter et al. 2023; Williams et al. 1999).

#### Control variables

We measured gender (0 = male, 1 = female), age, job position (0 = leadership, 1 = no leadership), education (1 = no education to 6 = university), and weekly working hours (1 = 0–9 to 6 = over 49) as sociodemographic variables. Disease-specific variables included self-stigma (Discrimination Experience subscale, ISMI; Boyd et al. 2014; 5 items, 4-point Likert scale, α = 0.90), disease duration (1 = < 1 year to 4 = > 5 years), disclosure to employer (0 = no, 1 = yes), treatment (0 = no, 1 = yes), and sickness absence due to MHI (1 = none to 6 = 100–365 days; Tuomi et al. 1997).

### Study-specific sample

*N* = 92 (9.5%) reported having a current MHI, whereas *N* = 877 (90.5%) reported no MHI. Table 1 displays the characteristics of the sample.

**Table 1:** Characteristics of the sample

	*MHI = Yes*		*MHI = No*	
	N	%	N	%
**Gender**				
* Male*	38	41.3	490	55.9
* Female*	54	58.7	386	44.0
**Age**	Mean = 48.7		Mean = 48.5	
**Education**				
* Basic education*	5	5.4	32	3.6
* Secondary education*	44	47.8	366	41.7
* High school diploma*	14	15.2	150	17.1
* University of applied sciences*	13	14.1	120	13.7
* University*	16	17.4	209	23.9
**Leadership position (yes)**	27	30	267	30.5
**Working hours/week**				
* 20–29 h*	11	12	83	9.5
* 30–39 h*	45	48.9	373	42.5
* 40–49 h*	34	37	412	47
* > 49 h*	2	2.2	9	1
**Received diagnosis (yes)**	77	83.7		
**Diagnosis**				
* Depression*	56	72.7		
* Anxiety disorder*	36	46.8		
* Psychosomatic disease*	18	23.4		
* Burnout*	16	20.8		
* Addiction disorder*	5	6.5		
* Other*	4	5.2		
**More than one diagnosis (yes)**	34	44.2		
**Duration of the disease**				
< 1 year	9	26.1		
1–3 years	24	26.1		
3–5 years	9	9.8		
> 5 years	50	54.3		
**Currrent treatment (psychotherapist or psychiatrist) (yes)**	48	52.2		
**Disclosure to the employer (yes)**	41	44.6		
**Sick leave**				
* Not within the last year*	45	48.9		
* 1–9 days*	13	14.1		
* 10–24 days*	22	23.9		
* 25–99 days*	5	5.4		
* 100–365 days*	7	7.6		

### Data analysis

The questionnaire was administered twice; however, for sociodemographic characteristics, disease-specific factors, and SOC, only data from Wave 1 were used. These variables were considered as relatively time-invariant for the respective time-frame. We first conducted correlation analyses to examine variable associations. Given the multilevel data structure (two measurement points per employee), mixed-effects models (also referred to as multilevel modeling, or hierarchical linear modeling) accounted for nesting due to the non-independence in the data structure, which violates assumptions of standard regression analyses (Byrk and Raudenbush 1992; Hox 2002). Specifically, we used a two-level structure with measurement occasions (Level 1) nested within individuals (Level 2). This approach makes efficient use of the available data, accounts for intra-individual correlation and allows for accurate statistical inference based on the repeated measures design.

Initial models included sociodemographic controls and mental health status as a fixed predictor, with burnout, work engagement, and work ability as outcomes. Subsequently, psychosocial working conditions SOC were added as additional predictors to investigate whether the effects of mental health status on occupational health outcomes remained significant after accounting for these variables. For employees with MHI, additional models included disease-specific controls, while psychosocial working conditions and SOC were predictors.

We also conducted a dropout analysis using multivariate logistic regression. Wave 1 study variables predicted participation in both waves. No significant differences emerged in main predictors or outcomes, but older participants were more likely to complete both waves (OR = 1.015, 95% CI [1.002, 1.029], *p* =.023). As attrition was unrelated to key variables, we ruled out systematic bias.

Due to some missing values, sample sizes varied slightly across analyses. Most models were based on *N* = 90 participants with MHI (143 observations) and *N* = 875 without MHI (1333 observations). Descriptive analyses used the full available data, with up to *N* = 92 with MHI (147 observations) and *N* = 877 without MHI (1335 observations).

## Results

The correlation analysis is displayed in Table 2 for the total sample and in Table 3 for employees with MHI.

**Table 2:** Intercorrelations of the variables of the whole sample

Variable	1	2	3	4	5	6	7	8	9	10
1. Age										
2. Gender	− 0.04									
3. Education	− 0.15**	− 0.03								
4. Job Position	0.03	0.11**	− 0.22**							
5. Working hours	− 0.13**	− 0.22**	0.15**	− 0.09**						
6. Job Resources	− 0.04	− 0.05	0.13**	− 0.16**	− 0.00					
7. Job Demands	− 0.16**	− 0.01	0.08*	− 0.04	0.12**	− 0.20**				
8. SOC	0.15**	− 0.10**	0.05	− 0.10**	− 0.01	0.38**	− 0.35**			
9. Work Engagement	0.07*	0.00	0.07*	− 0.18**	− 0.02	0.51**	− 0.19**	0.47**		
10. Burnout	− 0.18**	0.07*	− 0.01	0.09**	0.10**	− 0.46**	0.55**	− 0.61**	− 0.55**	
11. Work Ability	0.01	0.02	0.05	− 0.08*	0.00	0.31**	− 0.23**	0.45**	0.46**	− 0.49**

Note. *N* = 966–969 * *p* <.05, ** *p* <.01. Pearson correlation coefficients are reported for all variables except for the correlations involving the ordinal variables ‘education’ and ‘working hours’. Spearman correlation coefficients are reported for these correlations

**Table 3:** Intercorrelations of the variables among employees with MHI

Variable	1	2	3	4	5	6	7	8	9	10	11	12	13	14	15
1. Age															
2. Gender	− 0.23*														
3. Education	− 0.12	− 0.02													
4. Job Position	− 0.04	0.23*	− 0.32**												
5. Working hours	− 0.08	− 0.14	0.24*	− 0.21*											
6. Job Resources	0.01	− 0.06	− 0.02	− 0.03	− 0.07										
7. Job Demands	0.13	− 0.18	0.06	− 0.01	0.29**	− 0.40**									
8. SOC	0.13	− 0.01	− 0.01	− 0.05	0.04	0.51**	− 0.35**								
9. Work Engagement	0.02	0.08	− 0.03	0.05	− 0.03	0.55**	− 0.30**	0.49**							
10. Burnout	0.03	0.09	0.11	− 0.06	0.13	− 0.59**	0.60**	− 0.57**	− 0.54**						
11. Work Ability	− 0.14	0.26*	− 0.17	0.23*	− 0.19	0.42**	− 0.39**	0.38**	0.55**	− 0.50**					
12. Duration of the disease	− 0.01	0.17	− 0.12	0.15	− 0.16	0.10	− 0.13	− 0.03	− 0.05	− 0.06	0.16				
13. Treatment	0.12	− 0.18	− 0.02	− 0.04	− 0.07	− 0.11	0.20	− 0.15	− 0.12	0.17	− 0.15	0.67			
14. Disclosure	− 0.17	0.18	0.03	0.03	0.05	− 0.11	− 0.19	− 0.04	− 0.06	0.04	0.15	0.48	− 0.38**		
15. Sick leave	0.15	− 0.07	0.00	− 0.04	0.14	− 0.27**	0.27**	− 0.22*	− 0.19	0.38**	− 0.48**	0.49	0.31**	− 0.21*	
16. Self-stigma	− 0.15	− 0.03	− 0.03	− 0.02	0.05	− 0.31**	0.46**	− 0.34**	− 0.16	0.42**	− 0.34**	0.12	0.27*	− 0.24*	0.26*

Note. *N* = 90–92, * *p* <.05, ** *p* <.01. The numbers in brackets refer to the subsample of employees with MHI. Pearson correlation coefficients are reported for all variables except for the correlations involving the ordinal variables ‘education’, ‘working hours’, ‘duration of the disease’ and ‘sick leave’. Spearman correlation coefficients are reported for these correlations

The correlation analyses indicated that all predictors were associated with the outcomes in the expected direction. The results from the mixed-effects models are presented in Table 4 for testing Hypothesis 1 and in Table 5 for testing Hypotheses 2–4.

**Table 4:** Results of the mixed-effect models: occupational health among employees with and without MHI

	Burnout				Work Engagement					Work Ability				
	Model 1.1	Model 1.2	Model 1.3	Model 1.4	Model 2.1		Model 2.2	Model 2.3	Model 2.4	Model 3.1		Model 3.2	Model 3.3	Model 3.4
*Predictors*	*Estimates*	*Estimates*	*Estimates*	*Estimates*	*Estimates*		*Estimates*	*Estimates*	*Estimates*	*Estimates*		*Estimates*	*Estimates*	*Estimates*
Intercept	2.33 ^***^	2.94 ^***^	4.03 ^***^	4.12 ^***^	3.38 ^***^		2.95 ^***^	-0.85	-1.07 ^**^	7.53 ^***^		5.93 ^***^	2.50 ^***^	2.71 ^***^
Age	-0.02 ^***^	-0.01 ^***^	-0.01 ^**^	-0.01 ^**^	0.01 ^**^		0.01 ^**^	0.01†	0.01†	0.00		0.00	-0.01 ^*^	-0.01 ^*^
Gender	0.13 ^*^	0.10†	0.05	0.05	0.05		0.07	0.13†	0.14†	0.12		0.20†	0.29 ^**^	0.27 ^**^
Education	-0.02	-0.01	0.00		0.05		0.04	-0.01		0.05		0.03	-0.02	
Working Hours per Week	0.14 ^***^	0.15 ^***^	0.07 ^*^	0.07 ^*^	-0.11		-0.11	-0.04		-0.01		-0.03	0.05	
Job Position	0.17 ^**^	0.18 ^**^	0.06		-0.61 ^***^		-0.61 ^***^	-0.37 ^***^	-0.37 ^***^	-0.33 ^*^		-0.35 ^**^	-0.13	-0.13
MHI (Ref: current MHI)		-0.73 ^***^	-0.22 ^**^	-0.21 ^**^			0.53 ^***^	-0.13	-0.13			1.94 ^***^	1.13 ^***^	1.14 ^***^
Job Resources			-0.28 ^***^	-0.28 ^***^				0.74 ^***^	0.74 ^***^				0.49 ^***^	0.49 ^***^
Job Demands			0.42 ^***^	0.42 ^***^				-0.12 ^*^	-0.12 ^*^				-0.21 ^**^	-0.21 ^**^
SOC			-0.34 ^***^	-0.34 ^***^				0.44 ^***^	0.44 ^***^				0.64 ^***^	0.64 ^***^
**Random Effects**														
σ^2^	0.16	0.16	0.15	0.15	0.87		0.87	0.87	0.87	1.41		1.42	1.42	1.42
τ_00_	0.61 _ID_	0.56 _ID_	0.22 _ID_	0.22 _ID_	1.29 _ID_		1.27 _ID_	0.66 _ID_	0.66 _ID_	2.37 _ID_		2.04 _ID_	1.35 _ID_	1.35 _ID_
Observations	1476	1476	1476	1476	1476		1476	1476	1476	1476		1476	1476	1476
N	965	965	965	965	965		965	965	965	965		965	965	965
AIC	3353.135	3296.725	2574.786	2566.319	5137.697		5129.717	4769.727	4757.165	5931.473		5839.295	5608.275	5596.959

Note. †*p* <.10, * *p* <.05, ** *p* <.01, *** *p* <.001

**Table 5:** Results of the mixedeffect models: occupational health among employees with MHI

*Predictors*	Burnout				Work Engagement				Work Ability			
	Model 1.5	Model 1.6	Model 1.7	Model 1.8	Model 2.5	Model 2.6	Model 2.7	Model 2.8	Model 3.5	Model 3.6	Model 3.7	Model 3.8
	*Estimates*	*Estimates*	*Estimates*	*Estimates*	*Estimates*	*Estimates*	*Estimates*	*Estimates*	*Estimates*	*Estimates*	*Estimates*	*Estimates*
Intercept	1.16	-0.50	2.11 ^*^	2.55 ^**^	2.78	4.47 ^*^	-0.32	-0.70	6.76 ^*^	8.14 ^**^	2.91	1.98
Age	0.01	0.01	0.01		0.01†	0.01	0.01	-0.00	-0.03	-0.02	-0.03	
Gender	0.23	0.27	0.35 ^*^	0.34 ^*^	0.26	0.24	0.19		1.01†	0.82	0.73	0.89 ^*^
Working Hours per Week	0.32 ^*^	0.16	0.03	0.01	-0.12	-0.03	0.05		-0.39	0.06	0.11	
Job Position	-0.10	-0.13	-0.28†	-0.27†	-0.07	-0.02	0.13		0.85	0.91†	1.08 ^*^	1.02 ^*^
Duration of the Disease		-0.00	0.04			-0.14	-0.17			0.08	0.05	
Treatment		0.04	-0.01			-0.28	-0.23			0.24	0.34	
Disclosure		0.44 ^*^	0.27	0.25		-0.41	-0.08			-0.12	0.22	
Sickness absence		0.23 ^**^	0.13 ^*^	0.13 ^*^		-0.24	-0.07			-0.86 ^***^	-0.67 ^***^	-0.71 ^***^
Self-Stigma		0.47 ^***^	0.11	0.10		-0.20	0.20			-0.86 ^**^	-0.41	-0.41
Job Resources			-0.22 ^**^	-0.22 ^**^			0.53 ^***^	0.58 ^***^			0.39†	0.44 ^*^
Job Demands			0.47 ^***^	0.48 ^***^			-0.25				-0.23	
SOC			-0.34 ^***^	-0.33 ^***^			0.47 ^**^	0.52 ^***^			0.76 ^**^	0.73 ^**^
**Random Effects**												
σ^2^	0.19	0.19	0.14	0.14	0.66	0.67	0.63	0.65	1.15	1.14	1.16	1.19
τ_00_	0.87 _ID_	0.63 _ID_	0.35 _ID_	0.33 _ID_	1.95 _ID_	1.88 _ID_	1.28 _ID_	1.15 _ID_	5.98 _ID_	4.26 _ID_	3.35 _ID_	3.14 _ID_
Observations	143	143	143	143	143	143	143	143	143	143	143	143
N	90	90	90	90	90	90	90	90	90	90	90	90
AIC	376.1520	369.022	322.096	303.737	518.388	527.438	508.359	483.448	635.417	617.527	608.712	592.449

Note. †*p* <.10, * *p* <.05, ** *p* <.01, *** *p* <.001

### Differences in occupational health among employees with and without MHI

#### Burnout as outcome

In Model 1.1, socio-demographic variables were included, showing that younger individuals, women, those with longer working hours, and lower job positions had higher burnout levels. Model 1.2 added MHI, indicating that individuals with a current MHI reported more burnout, an effect that remained significant in Model 1.3 after including job demands, job resources, and SOC—though its impact was reduced. In Model 1.3, job resources and SOC were negatively, and job demands positively, associated with burnout. The final model (1.4) retained only significant variables (*p* <.10), with gender and job position no longer significant. The data showed that employees with a current MHI report higher levels of burnout compared to those without a MHI that supported Hypothesis 1a.

#### Work engagement as outcome

In Model 2.1, socio-demographic variables were included, showing that older individuals and those in higher job positions had higher work engagement. Model 2.2 added MHI, indicating lower work engagement for individuals with current MHI, though this effect was no longer significant in Model 2.3 after including job demands, job resources, and SOC. In this step, gender became significant (*p* <.10), indicating higher work engagement among women. The final model (2.4) retained only significant variables (*p* <.10), with age no longer significant. As MHI was not associated with work engagement, hypothesis 1b was rejected.

#### Work ability as outcome

In Model 3.1, socio-demographic variables were included, showing that higher job positions were associated with greater work ability. Model 3.2 added MHI, revealing lower work ability for individuals with current MHI compared to employees without MHI, an effect that remained significant in Model 3.3 after including job demands, job resources, and SOC—though its impact was reduced. In Model 3.3, job resources and SOC were positively, and job demands negatively, associated with work ability. Additionally, age and gender became significant, with older individuals and men reporting lower work ability. The final model (3.4) retained only significant variables (*p* <.10), with job position no longer significant. As employees with MHI reported lower work ability, hypothesis 1c was supported.

### The association between the psychosocial working conditions and SOC among employees with current MHI

To address Hypotheses 2–4, a subsample of employees with current MHI was selected. Psychosocial working conditions and SOC were included as predictors in the model. Additionally, disease-specific factors, along with sociodemographic variables as control variables, were accounted for (see Table 5).

#### Burnout as outcome

In Model 1.5, socio-demographic variables were included, with working hours significantly associated with burnout. Model 1.6 added disease-specific variables, showing that higher sickness absence and self-stigma were linked to higher burnout, and disclosure was associated with increased burnout. In Model 1.7, job resources and SOC were negatively, and job demands positively, associated with burnout. Gender also became significant, with women reporting higher burnout. When selecting only the significant predictors on a *p*-level < 0.10 from the two steps (model 1.8), we found significant associations between socio-demographic (gender), disease-specific (sickness absence), psychosocial working conditions (job resources and job demands), SOC and burnout. Thus hypotheses 2a, 2 and 4a were supported.

#### Work engagement as outcome

In Model 2.5, socio-demographic variables were included but showed no significant associations with work engagement. Model 2.6 added mental health-specific factors, which also showed no significant effects. In Model 2.7, job resources were positively associated with work engagement, while job demands were not significant. SOC was also significantly linked to work engagement in the expected direction. Thus, hypotheses H2b and H4b were supported.

#### Work ability as outcome

In Model 3.5, gender was significant (*p* <.10), with female employees reporting higher work ability. Model 3.6 added mental health-specific factors, showing that sickness absence and self-stigma were negatively associated with work ability, while job position (*p* <.10) indicated lower work ability for employees with leadership roles. Model 3.7 included psychosocial factors, revealing significant positive associations of job resources and SOC with work ability, while job demands were not significant. Model 3.8 retained only significant predictors (*p* <.10), supporting associations between gender, job position, sickness absence, job resources, and SOC with work ability. Thus, hypotheses H2c and H4c were supported.

### Post-hoc analyses

Given the significant role of psychosocial working conditions and SOC for employees with MHI, we tested whether these factors had a stronger impact on employees with MHI compared to those without MHI by adding interaction effects to the models in Table 4 (see Table 6).

**Table 6:** Results of the mixed-effect models: interaction effects between psychosocial working Conditions, SOC and MHI

*Predictors*	Burnout	Work Engagement	Work Ability	
	Model 1.9	Model 2.9	Model 3.9a	Model 3.9b
	*Estimates*	*Estimates*	*Estimates*	*Estimates*
Intercept	3.85 ^***^	-0.65	0.96	1.29
Age	-0.01 ^***^	0.01†	-0.01 ^*^	-0.01 ^*^
Gender	0.05	0.14†	0.27 ^**^	0.25 ^*^
Working Hours per Week	0.07 ^*^			
Job Position	0.06	-0.36 ^***^	-0.14	
MHI (Ref: Current MHI)	0.00	-0.63	3.22 ^***^	2.66 ^***^
Job Resources	-0.26 ^***^	0.59 ^***^	0.70 ^***^	0.49 ^***^
Job Demands	0.50 ^***^	-0.13 ^*^	-0.21 ^**^	-0.21 ^**^
SOC	-0.35 ^***^	0.46 ^***^	0.92 ^***^	0.99 ^***^
Job Resources * MHI	-0.01	0.18	-0.26	
Job Demands * MHI	-0.08			
SOC * MHI	0.01	-0.02	-0.31†	-0.39 ^*^
**Random Effects**				
σ^2^	0.15	0.87	1.43	1.43
τ_00_	0.22 _ID_	0.66 _ID_	1.32 _ID_	1.33 _ID_
Observations	1476	1476	1476	1476
N	965	965	965	965
AIC	2581.368	4763.961	5596.836	5592.092

Note. In the models the significant variables of the previous analyses (see Table 4) were entered

† *p* <.10, * *p* <.05, ** *p* <.01, *** *p* <.001

**Fig. 1:**
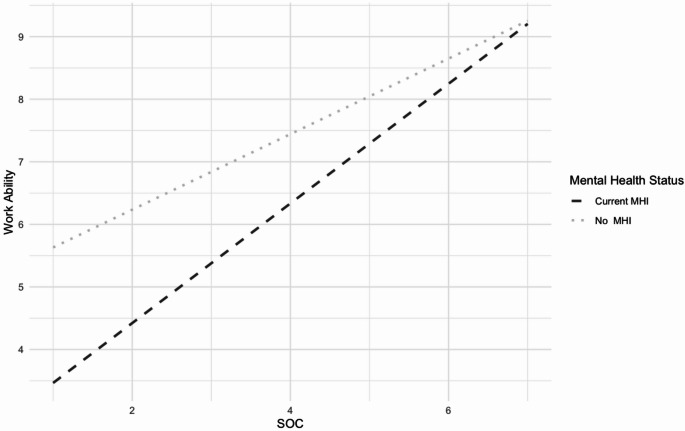
Interaction between mental health status and SOC on work ability

For burnout, no significant interaction effects of job demands, job resources, and SOC by MHI were found (Model 1.9). Similarly, no significant interaction effects for work engagement were found for job resources and SOC moderated by MHI (Model 2.9). For work ability, Model 3.9a revealed a marginally significant interaction between SOC and MHI (*p* <.10), which became statistically significant in Model 3.9b after removing non-significant predictors. Figure 1 shows predicted values from the mixed-effect model illustrating the interaction between SOC and MHI on work ability. It illustrates that SOC had a stronger impact on work ability for employees with MHI compared to those without MHI.

## Discussion

The aim of this study was to compare occupational health outcomes between employees with and without MHI and investigate factors associated with occupational health, particularly among employees with MHI. The findings show that employees with MHI report lower occupational health, particularly in terms of burnout and work ability, compared to employees without MHI. No significant difference in work engagement was found. To further examine factors influencing occupational health among employees with MHI, the findings highlight the crucial role of a combination of disease-specific, individual, and work-related factors. Notably, the study emphasizes the importance of SOC as an individual factor and job resources as a work-related factor, with both showing a positive association with all three occupational health outcomes. Previous research has already highlighted the role of SOC in health outcomes among the general working population (Vogt et al. 2016). Similarly, the predictive power of job resources for health outcomes has been observed in the general workforce, aligning with the JD-R framework, which links job resources to both motivating aspects of work (e.g., work engagement, work ability) and to reducing health-impairing processes (e.g., burnout) (Bakker et al. 2005).

Although our findings on predictors such as SOC and psychosocial working conditions align with previous research in the general population, population-specific mechanisms may also play a role. Disease-specific factors are likely to be significant, as individuals with MHI may have unique needs for specific job resources, which could be addressed through employee assistance programs or accommodations (Follmer and Jones 2018). While general population predictors remain relevant, their application and strategies may differ for employees with MHI.

Our study includes disease-specific control variables, such as sickness absence, which was identified as a key predictor of burnout and work ability. Despite the role of sickness absence as a disease-specific factor, it must be emphasized that the results particularly highlight the role of psychosocial working conditions and SOC. The post-hoc analyses further revealed that the relationship between mental health status and work ability is specifically moderated by SOC. This means that the impact of SOC on work ability is higher for employees with MHI compared to employees without MHI. This can be explained by the concept of SOC, which is an important life orientation for individuals who have faced adverse experiences (Antonovsky 1987).

### Implications

This study underscores the importance of supporting employees with MHI, who report lower occupational health (work ability and burnout). It highlights the key role of personal (e.g., SOC) and job resources in improving all three health outcomes. Strengthening job resources may require complex interventions (Nielsen et al. 2010), while individual-level interventions can positively influence SOC levels both generally (Kähonen et al., 2012; Vastamäki et al. 2009) and among those with MHI (Forsberg et al. 2010).

However, disease-specific factors remain relevant, particularly sickness absence, which affects work ability and burnout. While sickness absence is often used as an indicator of health status, its underlying meaning remains complex. It may reflect the acute severity of the illness and thus be a crucial predictor of an individual’s long-term ability to work (Virtanen et al. 2006). However, it might also serve as a coping strategy to prevent excessive strain or symptom deterioration (Van Rhenen et al. 2008). Furthermore, sickness absence could be influenced by workplace factors, such as the level of social support or job complexity (Väänänen et al. 2003).

### Limitations and future research

The main limitation of the study is its cross-sectional design, which prevents causal conclusions. However, previous studies have tested the antecedents of the JD-R model for causality (Lesener et al. 2019). Causality for SOC can also be inferred based on prior research (Heikinheimo et al. 2016). Nevertheless, these causal relationships should be replicated in future longitudinal studies within this specific population.

Another limitation might be that mental health is measured using a single self-report item, which may be prone to bias. However, one argument in favor of self-reports is that individuals typically have a good sense of their own mental state, making these measures both reliable and valid (Reme at al. 2010; Rutter et al. 2023; Williams et al. 1999). Additionally, self-reports require respondents to assign themselves a ‘mental illness identity’ (Follmer and Jones 2018), which enables the inclusion of disease-specific control variables.

Additionally, we did not investigate organizational disease-specific factors, such as employment assistance programs or accommodations. These may be covered by the general job resources construct, but it would be valuable for future studies to differentiate and explore the role of disease-specific components within job resources, potentially through qualitative or mixed-methods approaches.

Furthermore, the high number of measured variables in relation to the limited sample size among employees with MHI reduces the robustness of statistical inferences. However, the detection of significant effects in such a small sample suggests the presence of large effect sizes (Cohen 1992). At the same time, it is likely that smaller effects could not be detected due to limited statistical power. This warrants cautious interpretation, particularly regarding findings of statistical significance.

## Conclusion

To retain employees with MHI, it is crucial to support their work ability, prevent burnout, and foster engagement. Based on the JD-R model, this study examined validated work-related and personal factors. Findings show that psychosocial working conditions and SOC significantly impact these outcomes. Since both SOC and job resources are linked to all occupational health outcomes, strengthening them is essential for keeping employees with MHI engaged and productive.

## Data Availability

The data that support the findings of this study are available from the corresponding author upon reasonable request.
